# Loss of Genetic Diversity and Increased Subdivision in an Endemic Alpine Stonefly Threatened by Climate Change

**DOI:** 10.1371/journal.pone.0157386

**Published:** 2016-06-27

**Authors:** Steve Jordan, J. Joseph Giersch, Clint C. Muhlfeld, Scott Hotaling, Liz Fanning, Tyler H. Tappenbeck, Gordon Luikart

**Affiliations:** 1 Department of Biology, Bucknell University, Lewisburg, Pennsylvania, United States of America; 2 U.S. Geological Survey, Northern Rocky Mountain Science Center, Glacier National Park, West Glacier, Montana, United States of America; 3 Flathead Lake Biological Station, Montana Conservation Genomics Laboratory, Division of Biological Sciences, University of Montana, Polson, Montana, United States of America; 4 University of Kentucky, Department of Biology, Lexington, Kentucky, United States of America; University of Innsbruck, AUSTRIA

## Abstract

Much remains unknown about the genetic status and population connectivity of high-elevation and high-latitude freshwater invertebrates, which often persist near snow and ice masses that are disappearing due to climate change. Here we report on the conservation genetics of the meltwater stonefly *Lednia tumana* (Ricker) of Montana, USA, a cold-water obligate species. We sequenced 1530 bp of mtDNA from 116 *L*. *tumana* individuals representing “historic” (>10 yr old) and 2010 populations. The dominant haplotype was common in both time periods, while the second-most-common haplotype was found only in historic samples, having been lost in the interim. The 2010 populations also showed reduced gene and nucleotide diversity and increased genetic isolation. We found lower genetic diversity in *L*. *tumana* compared to two other North American stonefly species, *Amphinemura linda* (Ricker) and *Pteronarcys californica* Newport. Our results imply small effective sizes, increased fragmentation, limited gene flow, and loss of genetic variation among contemporary *L*. *tumana* populations, which can lead to reduced adaptive capacity and increased extinction risk. This study reinforces concerns that ongoing glacier loss threatens the persistence of *L*. *tumana*, and provides baseline data and analysis of how future environmental change could impact populations of similar organisms.

## Introduction

Climate change is rapidly altering physical and biological systems worldwide [1, 2]. Warming in the mid- to high-latitudes is occurring at two to three times the global average rate [3, 4] and in mountainous regions, particularly at higher-elevations, recent data show increased magnitude and rate of warming accompanied by extensive loss of glaciers and snowpack [5, 6]. These processes are having profound impacts on aquatic invertebrate communities in glacially fed, headwater streams [7, 8]. Substantial progress is being made in understanding how these species are likely to respond to climate warming, which is critical in developing conservation and recovery programs, yet much remains to be learned about the potential genetic effects of climate change on alpine stream biota, especially invertebrates [9–13]. The critical need to better understanding climate change’s genetic consequences is underscored by the fact that reductions in genetic diversity likely to accompany climate change also reduce the adaptive capacity, and thus evolutionary potential, of affected organisms.

The stonefly genus *Lednia* contains four species, all of which are known from high-elevation regions of the western United States [14, 15]. *Lednia tumana* (Plecoptera: Nemouridae) is endemic to the Waterton-Glacier International Peace Park region of Montana, Alberta, and British Columbia and is an obligate denizen of cold meltwater streams immediately below glaciers, permanent snowfields, and alpine springs [16]. Recent demographic surveys as well as climate and niche modeling suggest that the fate of *L*. *tumana* is likely tied to that of the region’s rapidly disappearing glaciers [5]; 125 of the estimated 150 glaciers existing in 1850 have disappeared, and the remaining 25 are predicted to be gone by 2030 if current warming trends continue. The loss of these glaciers and perennial snowfields is projected to reduce suitable habitat for *L*. *tumana* by over 80% (from ~23.2 to ~4.5 km^2^) of its predicted current range [16].

These major habitat reductions imply a greatly increased probability of extinction of *L*. *tumana* in the foreseeable future due to significant range contraction. As such, the species is currently a candidate for listing under the United States Endangered Species Act [17]. In many respects, *L*. *tumana* resembles *Zapada glacier* (Baumann and Gaufin), which is also in family Nemouridae, and occupies a similar range and habitats. Our recent work has found this species to be experiencing range contraction in the face of melting glaciers, with further population reductions predicted [18].

Here, we use analyses of mitochondrial DNA (mtDNA) sequences to explore population genetic structure and connectivity among 10 populations of *L*. *tumana*, and test the hypothesis that subdivision is increasing due to fragmentation over time. We chose to analyze mtDNA because it is easy to extract from historical samples (that were not collected or well preserved for DNA analyses), and because only mtDNA data exist for comparisons to related species. To help qualitatively test patterns of diversity and subdivision, we compare results to published mtDNA data from two other stonefly species, one from the same family and found in a high-latitude environment, and another from the same geographic region. Our data help clarify species and population status, inform endangered species listing considerations, and offer valuable insight into the potential impacts of climate warming on the genetic diversity of cold-climate freshwater invertebrates.

## Methods

**Fig 1:**
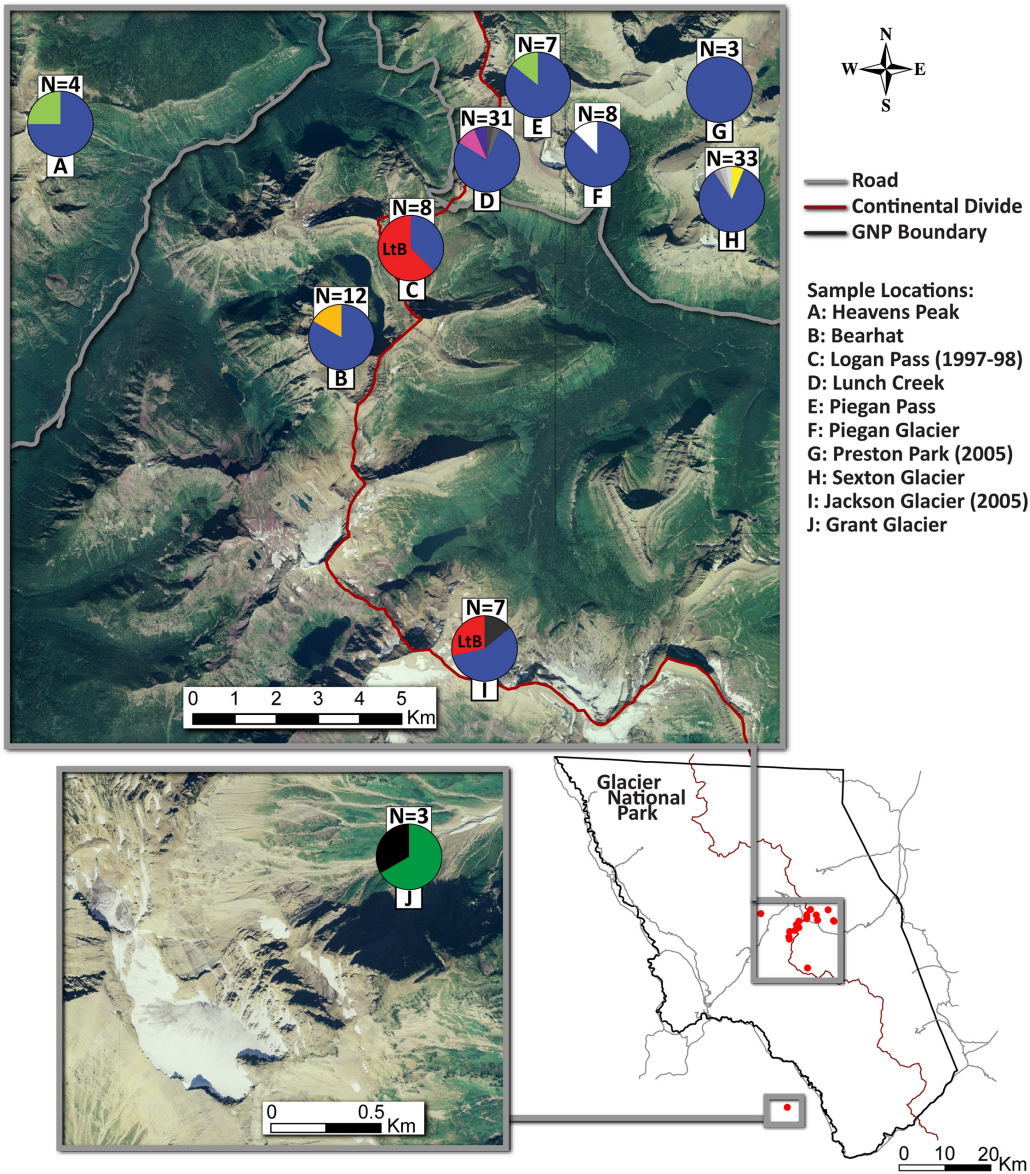
Sampling sites, sample sizes, and concatenated COI and CytB allele frequencies for *Lednia tumana* populations in the Glacier National Park region of Montana, USA. All sites without collecting dates were sampled in summer 2010. Individual haplotype colors correspond to those in Fig 2. Public domain imagery courtesy U. S. Department of Agriculture, Farm Service Agency.

We collected 98 nymphs and adults of *L*. *tumana* from Glacier National Park (GNP) and its environs in 2010 (Fig 1). These samples were supplemented with 18 ‘historic’ individuals collected in 1997, 1998, and 2005 (Table 1). All sampling was done under permit from the U.S. National Park Service. Samples were stored in 70–95% ethanol. The 16 sampled locations spanned the species’ known range, with locales separated by 0.3 to 44.3 km.

**Table 1:** Sampling and demographic information for 116 *Lednia tumana* individuals. Shaded boxes indicate adjacent sites that were pooled for combined analysis, and the Combined n refers to the combined totals of those sites.

		Combined					Larvae			Adults	
Location	n	n	Date	COI	CytB	undet	undet	Male	Female	Male	Female
**Mount Clements, east face** **Mount Clements, east face** **Logan Creek, nr. water tank** **Spring south of Reynolds Creek** **Below Reynolds Pass, east side**	3	8	6-Sep-97	1	3			1	2		
	2		6-Oct-98	1	1		2				
	1		3-Aug-98	0	1						1
	1		29-Jul-98	0	1				1		
	1		28-Jul-98	0	1						1
**Grant Glacier Basin**	3		6-Oct-10	1	3				1		2
**Heaven’s Peak, east face**	4		3-Sep-10	1	4			1	3		
**Hidden Lake, w. basin, s. face Bearhat** **Hidden Lake, w. basin** **Bearhat Mountain, east face**	1	12	7-Sep-10	1	1		1				
	6		7-Sep-10	4	6				3	1	2
	5		21-Aug-10	5	5			1	4		
**Jackson Glacier Basin**	7		12-Sep-05	5	7				2		5
**Lunch Creek, above high cliffs band** **Lunch Creek, below snow fields** **Lunch Creek, below cliff band** **Lunch Creek, side seep**	15	31	27-Aug-10	12	15		1	2	12		
	7		27-Aug-10	2	7			1	6		
	7		27-Aug-10	7	6			3	4		
	2		27-Aug-10	2	1				2		
**Stream West of Piegan Pass**	7		11-Sep-10	0	7				7		
**Piegan Glacier outlet** **E. face Mt. Piegan near Siyeh bend**	2	8	11-Sep-10	0	2				1		1
	6		25-Aug-10	4	6			1	3		2
**Above tarn at Preston Park**	3		14-Aug-05	0	3				3		
**Sexton Glacier Basin** **Sexton- South fork**	15	33	13-Sep-10	14	15	1		2	10		2
	18		13-Sep-10	17	14	1				14	3

We extracted DNA from legs, thoraces, or whole insects using DNEasy tissue kits (Qiagen, Hilden, Germany). We PCR-amplified two protein-coding mitochondrial genes, COI and CytB. COI primers were modified for this study from LCO1490 and HCO2198 of Folmer et al. [19]: F–TYTCAACAAATCAYAARGAYATTGG and R–tayacytcwggrtgmccaaaaaatca. CytB primers were taken from Kauwe et al. [20]: F–TGTCCATATTTGYCGAGATGT and R–CTTATGTTTTCAAAACATATGC. We used RedTAQ Genomic DNA Polymerase (Sigma-Aldrich, St. Louis, MO, USA) and the following conditions: 94° for 2 min, 35 cycles of 94° for 30 sec, either 58° (COI) or 50° (CytB) for 40 sec, and 72° for 60 sec, followed by 72° for 5 min and a 4° hold. PCR products were cleaned using a combination of Exonuclease I and Shrimp Alkaline Phosphatase. PCR products were Sanger sequenced in both directions using ABI 3730XL technology and the PCR primers. DNA sequences were corrected and aligned using CodonCode Aligner (CodonCode Corp., Centerville, MA, USA), concatenated using SequenceMatrix [21], and collapsed using Alter [22].

We explored for the presence of nuclear mitochondrial pseudogenes (numts) in our data using the recommendations from Fig 2 of Song et al [23]. We found no ghost bands in our agarose PCR gels and our sequences were generally very clean, with high quality scores and virtually no double peaks. Furthermore, our sequences contain no stop codons or nonsense mutations. BLAST searching confined to other Nemouridae species revealed no pairwise gaps and the vast majority of substitutions being at 1^st^ and 3^rd^ codon positions, suggesting strong stabilizing selection on a functional gene. Sequences have been deposited in GenBank (accession numbers KX212679-KX212864).

Molecular diversity measures including gene and nucleotide diversity and pairwise population *F*_*st*_ values were calculated using Arlequin 3.5 [24]. Gene diversity is roughly equivalent to the expected heterozygosity of diploid data, representing the probability that two randomly selected homologous haplotypes (alleles) from a population will be different. Nucleotide diversity is the probability that two randomly selected homologous nucleotides in the population will be different. Arlequin’s Fst calculations incorporate genetic differences between alleles. Geographic distance matrices were calculated using Geographic Distance Matrix Generator [25].

Arlequin analyses were run twice to test both fine- and coarse-scale genetic differentiation. First, we performed a within-stream analysis that kept all sampling sites separate to see if substantial fine-scale local (<2 km) genetic differentiation could be identified. For example, in Lunch Creek, we sampled four within-stream locations separated by 0.5 km or less. We calculated genetic diversity within and differentiation between each of these locations separately. Second, we performed within-basin analyses by combining sampling sites that were near each other (<2 km) as long as they were not separated by potential geographic barriers such as mountain ridges. This reduced the number of populations from 16 to 10 (Table 1). We also used Arlequin to run a Mantel test [26] of correlation between pairwise population F_st_ estimates and straight-line geographic distances. We ran the Mantel test for the seven 2010 populations only, using 100,000 permutations. We implemented AMOVA in Arlequin with each of the ten aggregated populations considered to be a group.

For qualitative comparisons of mtDNA diversity and subdivision with other species, we analyzed two previously published Plecoptera datasets using the same computational methods, including CytB data from *Pteronarcys californica* Newport (Pteronarcyidae) collected across the western United States, including mountainous regions [20] and COI data from *Amphinemura linda* (Nemouridae) collected near Churchill, Manitoba, Canada [27]. The *P*. *californica* dataset included 219 individuals from 27 populations, with sample sizes ranging from five to 10 individuals per population. Populations were separated from each other by between 16 and 978 km. The *A*. *linda* dataset included 179 individuals from three populations, with sample sizes ranging from three to 119 individuals. Populations were separated from each other by between 0.7 and 10.5 km.

In order to better compare allelic richness between populations and species, we used HP-Rare [28] to standardize population sample sizes. HP-Rare employs a rarefaction method to estimate allelic richness based on the smallest or other chosen sample size in a study. We ran the software on all populations with more than seven individuals from all three species. Total populations meeting or exceeding this threshold were seven from *L*. *tumana*, two from *A*. *linda*, and 23 from *P*. *californica*. We used SMOGD [29] to estimate values of G_ST_, G’_ST_, and D_est_ for both modern and historic populations, and to calculate 95% confidence ranges for these estimates.

## Results

**Fig 2:**
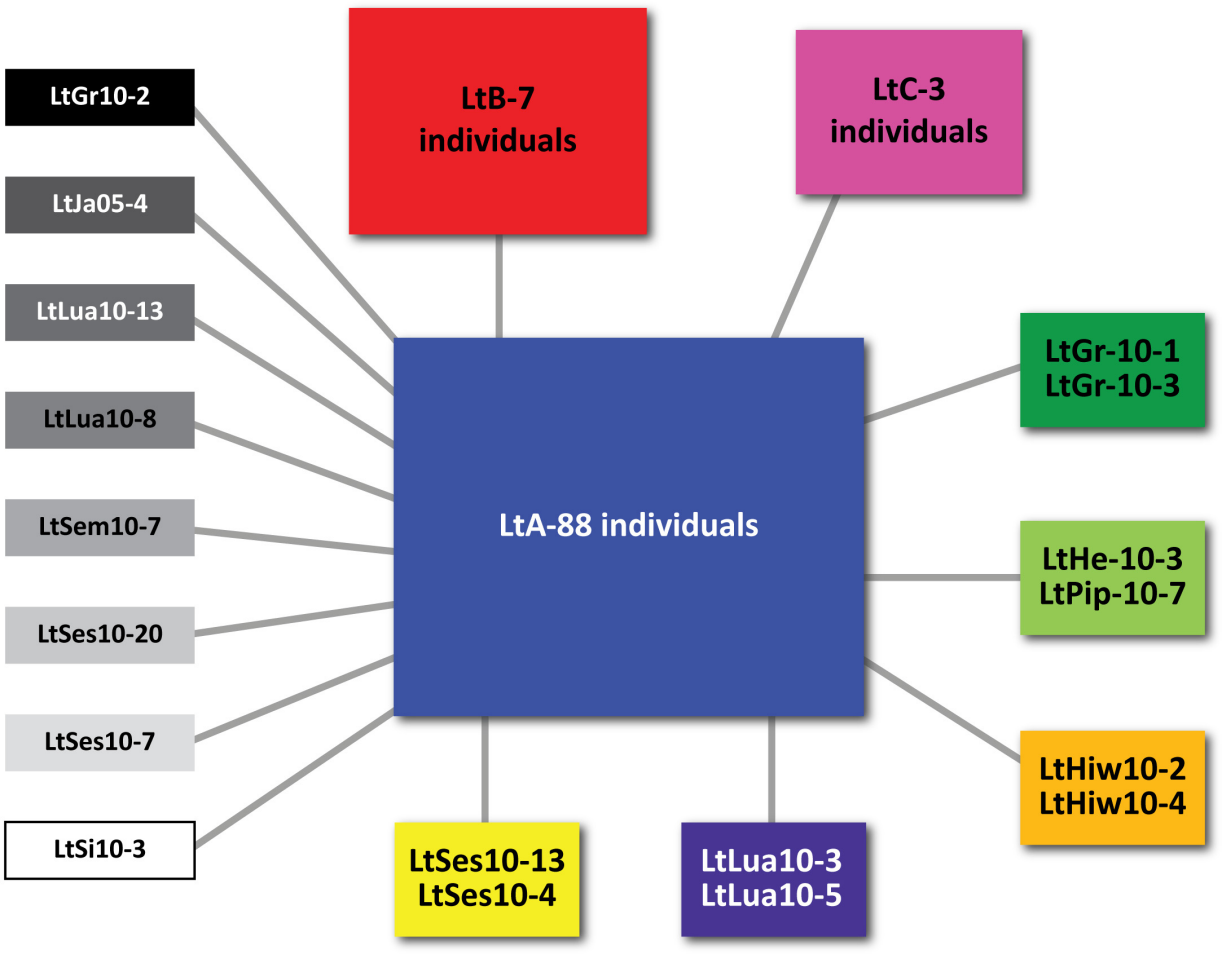
Minimum-spanning haplotype network for concatenated COI and CytB data from 116 *Lednia tumana* individuals. Individual haplotype colors correspond to those in Fig 1.

We obtained 658 bp of COI data and 872 bp of CytB data from 77 and 109 *L*. *tumana* individuals, respectively. The concatenated dataset had a total length of 1530 bp and 16 haplotypes. One of these haplotypes (LtA) was shared by 88 individuals, including many 2010 and historic samples. Each of the other 15 haplotypes differed from this dominant haplotype by only one substitution (Fig 2), suggesting again that our sequences do not contain numts. The second-most-common haplotype (LtB) was shared by seven historic individuals (from 1997, 1998, and 2005) from two populations separated by ~9km, but was absent from 2010 samples. This haplotype differs from LtA by a third codon position substitution near the end of the CytB gene (AA 351/378:Valine). While none of the 2010 samples were collected from the identical locations that harbored LtB, extensive sampling was done in 2010 within three km of the LtB sites (at Lunch Creek, for example). Of the remaining haplotypes, one was carried by three individuals, five were carried by two individuals, and eight were carried by single individuals.

**Fig 3:**
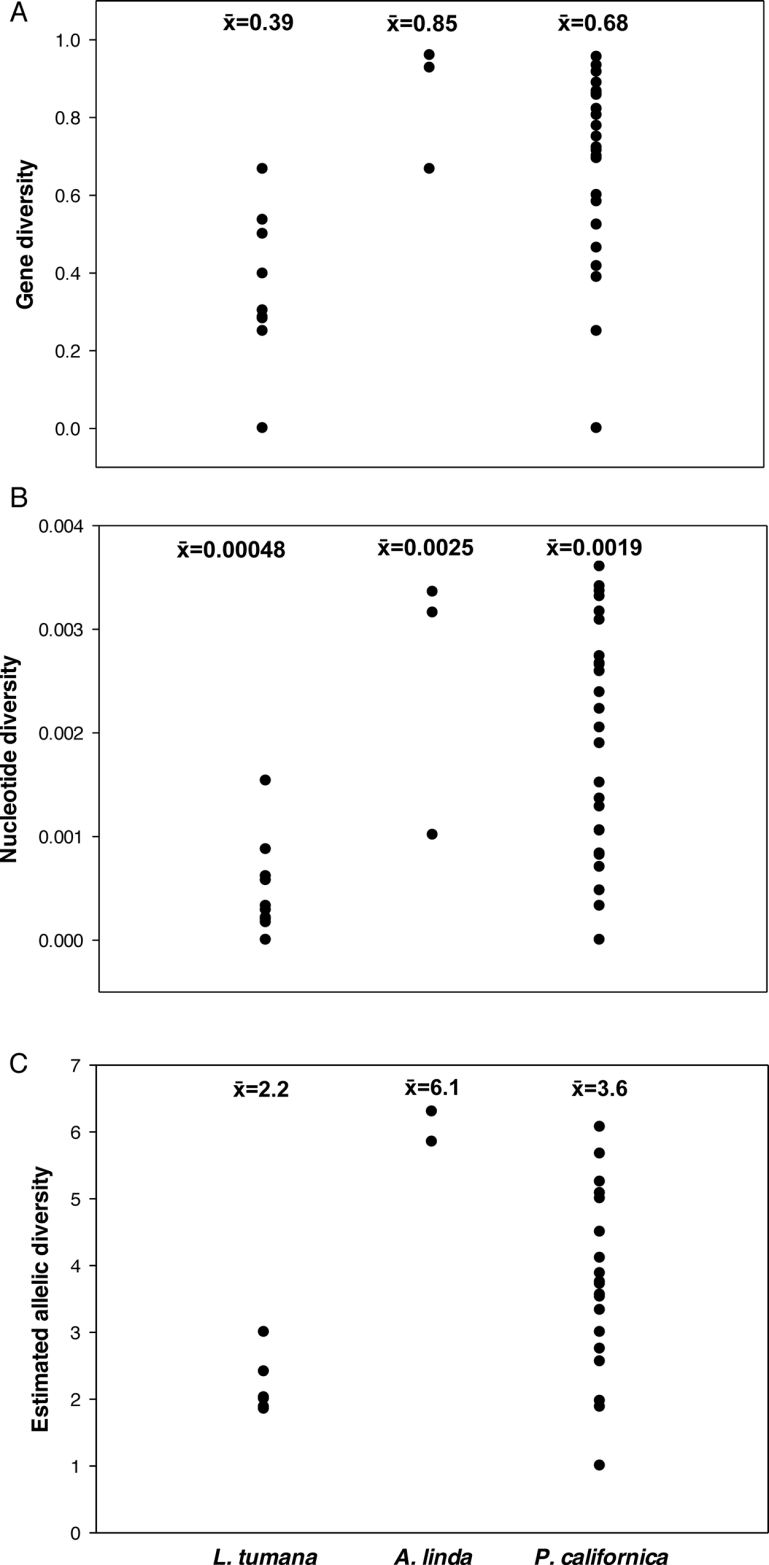
**Gene (A), nucleotide (B), and allelic (C) diversity for populations of three species of Plecoptera.** Allelic diversity was estimated using the rarefaction method implemented in HP-RARE [28].

Mean nucleotide and gene diversities in *L*. *tumana* were 0.00048 and 0.39, respectively (Fig 3), lower than those in both *A*. *linda* (0.0025 and 0.85; Fig 3) and *P*. *californica* (0.0019 and 0.68; Fig 3). Allelic richness estimates from HP-RARE were also lower in *L*. *tumana* than in *A*. *linda* and *P*. *californica*, with mean values of 2.2, 6.1, and 3.6, respectively (Table 2, Fig 3).

**Table 2:** Summary of results from population genetic analyses.

	Arlequin	HP-RARE	SMOGD				
	Global Fst	Mean Pop. Allelic Diversity	Hs_est	Ht_est	G_est	G_Hedrick	D_est
***Lednia tumana***	0.32	2.2	0.618	0.631	0.021	0.059	0.039
**2010 pops**	0.21		0.605	0.626	0.034	0.093	0.062
**Historic pops**	0.06		0.640	0.639	-0.003	-0.012	-0.007
***A*. *linda***	-0.02	6.1					
***P*. *californica***	0.24	3.6					

Pairwise *F*_*st*_ values were all effectively zero for the within-stream comparisons along Lunch Creek, and very low at other locations. For example, the *F*_*st*_ between the two Sexton populations (n = 15 and 18), which were separated by only 130 m, was 0.026, and was not statistically significant (p>0.05), meaning there was no population structure within streams. These results justified our approach of combining sampling locales within a few km of each other along the same creek or basin (e.g., for coarse-scale tests of population genetic subdivision).

AMOVA analysis of *L*. *tumana* revealed an overall *F*_*st*_ of 0.32 (Table 2), with 19.4% of variation among groups (populations) and 68.3% within populations. The mean of all pairwise *F*_*st*_ values between *L*. *tumana* populations (*F*_*st*_ = 0.19) was intermediate compared to that for the more widespread *P*. *californica* (0.35) and localized *A*. *linda* (-0.07), while the overall F_*st*_ of *L*. *tumana* was higher than the other two species (Table 2). The mean of nine *F*_*st*_ values of *P*. *californica* populations separated by 16 to 46 km was 0.12, with individual pairwise population comparisons ranging from 0 to 0.68 (the highest, outlying value being between the Yellowstone and Clark’s Fork River). The mean *F*_*st*_ between historic *L*. *tumana* population samples and 2010 samples was 0.23.

**Fig 4:**
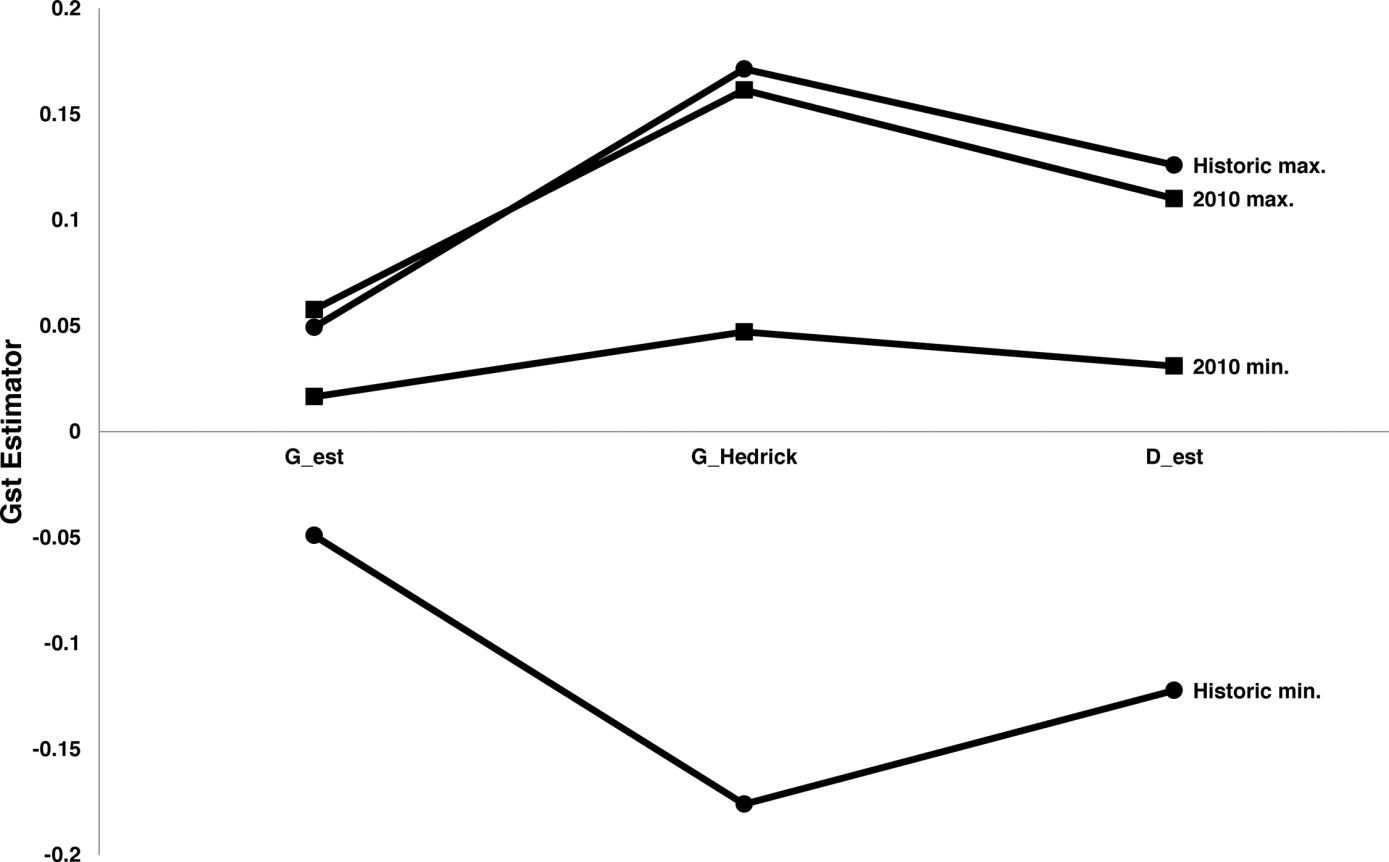
Estimates of G_st_ analog 95% confidence intervals from SMOGD [29] for historic and 2010 populations of *L*. *tumana*.

The Mantel test was highly significant (p = 0.01), with a correlation coefficient between the matrices of 0.91. The SMOGD calculations of 95% confidence intervals of G_ST_, G’_ST_, and D_est_ for modern and historic populations revealed nearly complete overlap between these intervals, failing to reject the hypothesis that by these metrics, diversity has remained constant over time (Fig 4).

## Discussion

### Genetic Diversity

The results of our population genetic analyses are consistent with those found in a small but growing body of literature addressing genetic diversity and divergence in alpine-adapted aquatic invertebrates threatened by climate change. Such studies suggest that the loss of alpine glaciers is causing significant declines in both taxonomic [10] and genetic [13] diversity as headwater environmental diversity declines. *Lednia tumana* has a restricted geographic and environmental range, being found in isolated sky-island populations in one of the most mountainous areas of North America, the Crown of the Continent Ecosystem [16, 30]. This species is further isolated by its relatively narrow habitat preferences that limit larvae to stream reaches that are typically less than 1 km from a glacial or permanent snowfield sources, and caves.

Extant *L*. *tumana* populations bear the signature of recent declines in genetic diversity. Mean nucleotide and gene diversity for historic samples were higher than those for the 2010 samples (7.4 x 10^−4^ vs. 2.4 x 10^−4^ and 0.60 vs. 0.30, respectively, including only populations with n>6). Furthermore, in spite of extensive sampling, we found no LtB haplotypes in 2010 populations, including many that were within 3 km of historic sampling sites where LtB was found. Additionally, historic populations had higher mean allelic richness than 2010 populations (2.5 vs. 2.0). This apparent loss of genetic diversity in *L*. *tumana* is consistent with small effective population sizes, which can lead to reduced fitness and evolutionary potential and increased genetic drift and extinction risk [31–33].

Both metrics of genetic diversity were lower in *L*. *tumana* than in either of the comparison species. Mean gene and nucleotide diversity estimates were two to five times lower (Fig 3). Likewise, mean allelic richness in *L*. *tumana* (2.2) was much lower than in *P*. *californica* (3.6) and *A*. *linda* (6.1; Fig 3). Because these estimates were standardized to a population sample size of seven for all three species using HP-Rare [28], they are perhaps the best interspecific comparison. When evaluating these results, it should be noted that CytB (used in comparisons with *P*. *californica*) is generally considered to be a more variable locus than COI [34].

### Gene Flow and Fragmentation

As species’ ranges are reduced and fragmented, we expect to see increased population isolation as indicated by decreased gene flow [35]. Our temporal and interspecific comparisons suggest that this may be the case for *L*. *tumana* over our 13-year study period. The mean *F*_*st*_ value between historic *L*. *tumana* populations and modern populations was 0.23, a value influenced by the presence of the LtB allele in the historic samples and suggesting substantial genetic change over time. Similarly, mean *F*_*st*_ values between historic *L*. *tumana* populations was 0.14, while the mean F_st_ among 2010 populations was 0.18, suggesting a possible decrease in gene flow and/or local effective population size. Rapidly changing genetic composition, reduced gene flow, and loss of haplotypes suggest that *L*. *tumana* is vulnerable to extinction.

Fragmentation, range contraction and rapid genetic change are increasingly problematic in many species [36]. Our data are consistent with the pattern described by Finn et al. [13] who used a space-for-time approach to compare mtDNA genetic diversity in the mayfly *Baetis alpinus* across a range of stream “glacialities” on the Iberian Penninsula. They found significantly lower regional genetic diversity in recently deglaciated streams compared to those with “high glaciality” characteristics. Similar findings have emerged in other systems as well. For instance, reductions in genetic diversity have also been identified in an obligate alpine chipmunk (*Tamias alpinus*) that has experienced documented, climate-change-induced range contraction in Yosemite National Park [37].

Our interspecific comparisons of stonefly species also suggest reduced gene flow and higher fragmentation in *L*. *tumana*. For example, the two well-sampled populations of *A*. *linda*, Ramsay Creek (n = 119) and Eastern Creek (n = 57), are separated by only 10.1 km, a value comparable to many of the *L*. *tumana* sites in this study. The terrain around Churchill has few dispersal barriers, being quite flat, and having a great deal of suitable intervening habitat. It is therefore not surprising that the *F*_*st*_ value between these *A*. *linda* populations was effectively zero, indicating panmixia. The mean pairwise *F*_*st*_ value from the combined analysis among 2010 *L*. *tumana* populations with similar separation (<17 km) was higher at 0.04, and may be the result of partial isolation, reduced gene flow, or a combination of the two, perhaps due to more mountainous terrain. This higher mean *F*_*st*_ also suggests potentially lower gene flow and higher fragmentation among modern *L*. *tumana* populations compared to *A*. *linda*.

The analyzed populations of *P*. *californica* were widely distributed across the western US, but nine were found within 50 km of each other in mountainous regions, conditions similar to those of our study. The mean *F*_*st*_ of these populations was 0.12, which was lower than the mean *F*_*st*_ value of 0.18 for all modern *L*. *tumana* populations (including the Grant Glacier basin population that is roughly 40 km south of the sampling core of this study). This suggests that genetic differentiation in *L*. *tumana* may be influenced by more than just terrain. These additional influences may include fragmentation associated with ice and snow loss and differing species vagility. Furthermore, although we only included three individuals from the Grant Glacier population, they carried two alleles not seen elsewhere, suggesting that over such scales *L*. *tumana* may be genetically divergent or diverging rapidly.

Our data join a small but growing body of data from studies seeking to understand the genetic effects of climate-change-induced glacier and snow loss on rare alpine macroinvertebrates. We found relatively low diversity within populations and high differentiation between populations of *L*. *tumana*, which is indicative of low connectivity among populations. This suggests that the likelihood of recolonization and genetic rescue as a means to mitigate climate-change-induced threats is relatively limited in *L*. *tumana*. Our genetic data are also consistent with previous studies indicating that *L*. *tumana* will likely continue to experience increased fragmentation and isolation, and decreased population size, due to the ongoing loss of suitable cold water alpine streams driven by climate warming [16]. Such predictions are further bolstered by a recent study of *Zapada glacier*, a nemourid stonefly historically found throughout GNP in similar habitats to *L*. *tumana*. This species has experienced a dramatic reduction in its occupied range in recent years [18].

The link between our genetic data, the fundamental niche of *L*. *tumana* (Muhlfeld et al. 2011), and regional climate change are relevant to current proposals to list *L*. *tumana* as endangered under the U.S. Endangered Species Act. If listing occurs, *L*. *tumana* could be the first invertebrate species listed as threatened or endangered due to climate change, and likely represents a guild of species facing similar threats in alpine headwaters worldwide.

## References

[1] Parmesan C, Yohe G. A globally coherent fingerprint of climate change impacts across natural systems. Nature. 2003;421: 37–42. 12511946 10.1038/nature01286

[2] Walther GR, Post E, Convey P, Menzel A, Parmesan C, Beebee TJ, et al Ecological responses to recent climate change. Nature. 2002;416: 389–395. 11919621 10.1038/416389a

[3] Pederson GT, Graumlich LJ, Fagre DB, Kipfer T, Muhlfeld CC. A century of climate and ecosystem change in Western Montana: what do temperature trends portend? Clim Change. 2010;98: 133–154.

[4] Hansen J, Nazarenko L, Ruedy R, Sato M, Willis J, Del Genio A, et al Earth's energy imbalance: confirmation and implications. Science. 2005;308: 1431–1435. 15860591 10.1126/science.1110252

[5] Hall M, Fagre D. Modeled climate-induced glacier change in Glacier National Park, 1850–2100. Bioscience. 2003;53: 131–140.

[6] Rauscher SA, Pal JS, Diffenbaugh NS, Benedetti MM. Future changes in snowmelt-driven runoff timing over the western US. Geophys Res Lett. 2008;35: L16703.

[7] Finn FS, Räsänen K, Robinson CT. Physical and biological changes to a lengthening stream gradient following a decade of rapid glacial recession. Global Change Biol. 2010;16: 3314–3326.

[8] Slemmons KEH, Saros JE, Simon K. The influence of glacial meltwater on alpine aquatic ecosystems: a review. Environ Sci: Processes Impacts. 2013;15: 1794–1806. 10.1039/c3em00243h 24056713

[9] Bálint M, Domisch S, Engelhardt C, Haase P, Lehrian S, Sauer J, et al Cryptic biodiversity loss linked to global climate change. Nature Climate Change. 2011;1: 313–318.

[10] Jacobsen D, Milner AM, Brown LE, Dangles O. Biodiversity under threat in glacier-fed river systems. Nature Climate Change. 2012;2: 361–364.

[11] Finn DS, Alder PH. Population genetic structure of a rare high-elevation black fly, *Metacnephia coloradensis*, occupying Colorado lake outlet streams. Freshwat Biol. 2006;51: 2240–2251.

[12] Finn DS, Theobald DM, Black WC, Poff NL. Spatial population genetic structure and limited dispersal in a Rocky Mountain alpine stream insect. Mol Ecol. 2006;15: 3553–3566. 17032257 10.1111/j.1365-294X.2006.03034.x

[13] Finn DS, Zamora-Munoz C, Murria C, Sainz-Bariain M, Alba-Tercedor J. Evidence from recently deglaciated mountain ranges that *Baetis alpinus* (Ephemeroptera) could lose significant genetic diversity as alpine glaciers disappear. Freshwater Science. 2014;33: 207–216.

[14] Baumann RW, Kondratieff BC. The stonefly genus *Lednia* in North America (Plecoptera: Nemouridae). Illiesia. 2010;6: 315–327.

[15] Baumann RW, Call RG. *Lednia tetonica*, a new species of stonefly from Wyoming (Plecoptera: Nemouridae). Illiesia. 2012;8: 104–110.

[16] Muhlfeld C, Giersch JJ, Hauer FR, Pederson G, Luikart G, Peterson D, et al Climate change links fate of glaciers and an endemic alpine invertebrate. Clim Change. 2011;106: 337–345.

[17] Fish US and Service Wildlife. Endangered and Threatened Wildlife and Plants. Federal Register. 2012;77: 69994–70060.

[18] Giersch JJ, Jordan S, Luikart Gordon, Jones LA, Hauer FR, Muhlfeld CC. Climate-induced range contraction of a rare alpine aquatic invertebrate. Freshwater Science. 2015;34: 53–65.

[19] Folmer O, Black M, Hoeh W, Lutz R, Vrijenhoek R. DNA primers for amplification of mitochondrial cytochrome c oxidase subunit I from diverse metazoan invertebrates. Mol Marine Biol Biotechnol. 1994;3: 294–299. 7881515

[20] Kauwe J, Shiozawa D, Evans R. Phylogeographic and nested clade analysis of the stonefly *Pteronarcys californica* (Plecoptera: Pteronarcyidae) in the western USA. J N Am Benthol Soc. 2004;23: 824–838.

[21] Vaidya G, Lohman DJ, Meier R. SequenceMatrix: concatenation software for the fast assembly of multi-gene datasets with character set and codon information. Cladistics. 2011;27: 171–180. 10.1111/j.1096-0031.2010.00329.x 34875773

[22] Glez-Pena D, Gomez-Blanco D, Reboiro-Jato M, Fdez-Riverola F, Posada D. ALTER: program-oriented conversion of DNA and protein alignments. Nucleic Acids Res. 2010;38 (suppl. 2): W14–W18. 20439312 10.1093/nar/gkq321 PMC2896128

[23] Song H, Buhay JE, Whiting MF, Crandall KA. Many species in one: DNA barcoding overestimates the number of species when nuclear mitochondrial pseudogenes are coamplified. Proceedings of the National Academy of Sciences. 2008;105: 13486–13491. 10.1073/pnas.0803076105 PMC2527351 18757756

[24] Excoffier L, Lischer HEL. Arlequin suite ver 3.5: a new series of programs to perform population genetics analyses under Linux and Windows. Molecular Ecology Resources. 2010;10: 564–567. 10.1111/j.1755-0998.2010.02847.x 21565059

[25] Ersts PJ. Geographic Distance Matrix Generator (version 1.2.3). Available from biodiversityinformatics.amnh.org/open_source/gdmg: American Museum of Natural History, Center for Biodiversity and Conservation; 2013.

[26] Mantel N. Detection of disease clustering and a generalized regression approach. Cancer Res. 1967;27: 209–220. 6018555

[27] Zhou X, Adamowicz S, Jacobus L, DeWalt RE, Hebert P. Towards a comprehensive barcode library for arctic life—Ephemeroptera, Plecoptera, and Trichoptera of Churchill, Manitoba, Canada. Frontiers in Zoology. 2009;6: 30 10.1186/1742-9994-6-30 20003245 PMC2800108

[28] Kalinowski ST. HP-RARE 1.0: a computer program for performing rarefaction on measures of allelic richness. Molecular Ecology Notes. 2005;5: 187–189.

[29] Crawford NG. SMOGD: software for the measurement of genetic diversity. Molecular Ecology Resources. 2010;10: 556–557. 10.1111/j.1755-0998.2009.02801.x 21565057

[30] Hauer FR, Stanford JA, Lorang MS. Pattern and process in Northern Rocky Mountain headwaters: Ecological linkages in the headwaters of the Crown of the Continent. J Am Water Resour Assoc. 2007;43: 104–117.

[31] Reed DH, Frankham R. Correlation between Fitness and Genetic Diversity; Correlación entre Adaptabilidad y Diversidad Genética. Conserv Biol. 2003;17: 230–237.

[32] Spielman D, Brook BW, Frankham R. Most species are not driven to extinction before genetic factors impact them. Proc Natl Acad Sci U S A. 2004;101: 15261–15264. 15477597 10.1073/pnas.0403809101 PMC524053

[33] DeSalle R. Conservation genetics—Genetics at the brink of extinction. Heredity. 2005;94: 386–387. 15674381 10.1038/sj.hdy.6800641

[34] Simon C, Frati F, Beckenbach A, Crespi B, Liu H, Flook P. Evolution, weighting, and phylogenetic utility of mitochondrial gene sequences and a compilation of conserved polymerase chain reaction primers. Annals of the Entomological Society of America. 1994;87: 651–701.

[35] Allendorf FW, Luikart G. Conservation and the Genetics of Populations Malden, MA: Wiley-Blackwell; 2007.

[36] Pauls SU, Nowak C, Bálint M, Pfenninger M. The impact of global climate change on genetic diversity within populations and species. Mol Ecol. 2013;22: 925–946. 10.1111/mec.12152 23279006

[37] Rubidge EM, Patton JL, Lim M, Burton AC, Brashares JS, Moritz C. Climate-induced range contraction drives genetic erosion in an alpine mammal. Nature Climate Change. 2012;2: 285–288.

