# Correction: Loss of Genetic Diversity and Increased Subdivision in an Endemic Alpine Stonefly Threatened by Climate Change

**DOI:** 10.1371/journal.pone.0159931

**Published:** 2016-07-19

**Authors:** Steve Jordan, J. Joseph Giersch, Clint C. Muhlfeld, Scott Hotaling, Liz Fanning, Tyler H. Tappenbeck, Gordon Luikart

## Notice of Republication

This article was republished on July 8, 2016, to correct shading errors in Table 1 and to remove information intended only for the draft version of the manuscript. Please download this article again to view the correct version.
